# Prediction of Protein Function from Tertiary Structure of the Active Site in Heme Proteins by Convolutional Neural Network

**DOI:** 10.3390/biom13010137

**Published:** 2023-01-09

**Authors:** Hiroko X. Kondo, Hiroyuki Iizuka, Gen Masumoto, Yuichi Kabaya, Yusuke Kanematsu, Yu Takano

**Affiliations:** 1 Faculty of Engineering, Kitami Institute of Technology, 165 Koen-cho, Kitami 090-8507, Japan; 2 Graduate School of Information Sciences, Hiroshima City University, 3-4-1 Ozukahigashi Asaminamiku, Hiroshima 731-3194, Japan; 3 Laboratory for Computational Molecular Design, RIKEN Center for Biosystems Dynamics Research, 6-2-3 Furuedai, Suita 565-0874, Japan; 4 Graduate School of Information Science and Technology, Hokkaido University, Kita 14, Nishi 9, Kitaku, Sapporo 060-0814, Japan; 5 Information Systems Division, RIKEN Information R&D and Strategy Headquarters, 2-1 Hirosawa, Wako 351-0198, Japan; 6 Graduate School of Advanced Science and Engineering, Hiroshima University, 1-4-1 Kagamiyama, Higashi-Hiroshima 739-8527, Japan

**Keywords:** structure–function correlation, active site conformation, convolutional neural network, machine learning

## Abstract

Structure–function relationships in proteins have been one of the crucial scientific topics in recent research. Heme proteins have diverse and pivotal biological functions. Therefore, clarifying their structure–function correlation is significant to understand their functional mechanism and is informative for various fields of science. In this study, we constructed convolutional neural network models for predicting protein functions from the tertiary structures of heme-binding sites (active sites) of heme proteins to examine the structure–function correlation. As a result, we succeeded in the classification of oxygen-binding protein (OB), oxidoreductase (OR), proteins with both functions (OB–OR), and electron transport protein (ET) with high accuracy. Although the misclassification rate for OR and ET was high, the rates between OB and ET and between OB and OR were almost zero, indicating that the prediction model works well between protein groups with quite different functions. However, predicting the function of proteins modified with amino acid mutation(s) remains a challenge. Our findings indicate a structure–function correlation in the active site of heme proteins. This study is expected to be applied to the prediction of more detailed protein functions such as catalytic reactions.

## 1. Introduction

Proteins with metal cofactors and ions are called metal proteins, where a metal ion and its environment work as a catalytic active center. Because metal proteins enable biochemical reactions not possible with ordinary proteins, many researchers pay attention to them [1,2,3,4,5]. Heme proteins are the largest class of metal proteins and serve pivotal biological functions. Heme, a Fe–porphyrin complex, is an active center of heme proteins and expresses diverse functions such as an electron transport [6,7], a catalyst for various kinds of reactions [8,9], and an oxygen carrier [10,11]. Besides being an active center, it plays a role in the regulation of protein functions as a ligand [12,13] and in a source of Fe ions [14]. Some proteins bind to heme for transport or storage; these are referred to as hemophores [15]. The mechanism of heme protein functions has been a crucial scientific issue. The structural information on heme proteins is increasing yearly [16], indicating a high level of scientific interest. However, few studies have comprehensively investigated heme proteins.

The key factors regulating the heme function are considered to be the axial ligand of heme, the side-chain orientation of the heme propionate, the types of heme, and the porphyrin distortion of heme. Because distal and proximal amino acids and chemical structures of heme are important factors in determining protein functions, their roles have been investigated [17,18,19]. However, these factors alone do not determine protein functions [20].

Both experimental and computational studies have shown a correlation between heme distortion and its chemical properties [21,22,23,24]. Our group discovered a heme distortion classified into oxygen-binding proteins and oxidoreductases by a combined analysis of machine learning and quantum chemical calculations [25]. Therefore, we focused on the contribution of heme distortion to the functional regulation of heme proteins. Heme complexed with its host protein exhibits a distorted conformation from its isolated structure [20], suggesting the regulation of the heme porphyrin structure by the protein environment around heme. From a simulation study for two oxygen carrier proteins, hemoglobin and myoglobin, it was suggested that the host protein environment affects heme distortion and controls chemical properties of heme relevant to the function of its host protein [26]. As a first step in clarifying such regulation of function of heme, we elucidated the correlations between the heme distortion and protein environment around heme, including proximal and distal amino acids using a machine learning method [27] and a convolutional neural network (CNN) [28]. Since the heme distortion correlates with its chemical properties, it is likely that it also correlates with protein function. Considering these results, we can expect to predict protein functions from the tertiary structures of heme-binding sites, including axial ligand(s).

In experimental studies, researchers are actively working on modifying the function of proteins by introducing amino acid mutations. Especially for myoglobin, which is an oxygen carrier, engineered proteins, such as peroxidase [29,30,31,32,33], exhibit enzymatic activity. These mutated sites are primarily located in heme-binding pockets (active sites) other than axial ligands. Thus, changes in the protein environment of a heme-binding site significantly affect protein functions.

In this study, we constructed a CNN model for predicting protein functions from the tertiary structures of the heme-binding sites of heme proteins, including proximal and distal amino acids. The CNN is a kind of deep neural network that is widely utilized in computer vision tasks, such as image classification [34,35]; it has also been applied to the classification of protein cavity structures [36]. We succeeded in predicting protein functions from the pocket structures of three functional groups of heme proteins. The prediction with our CNN model worked well between the groups with quite different functions. Analysis of the similarity of cavity shape among proteins with the same function suggests that there is no one-to-one correspondence between a protein function and a pocket structure. This study is expected to be applied to the prediction of more detailed protein functions such as catalytic reactions. This is the first step toward understanding structure–function relationships in the active sites of heme proteins.

## 2. Materials and Methods

### 2.1. Data Collection of Heme and Its Host Proteins

**Figure 1:**
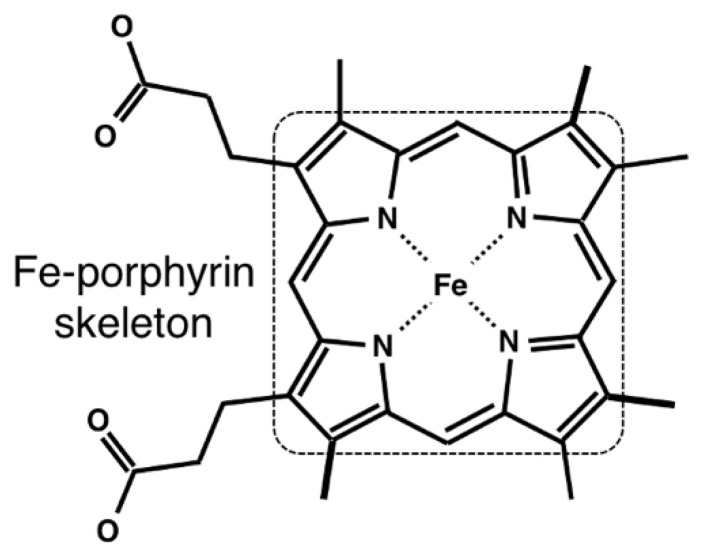
Chemical structure of heme. Fe–porphyrin skeleton is enclosed by a square-dotted line.

To collate the structural and functional information of heme proteins, we searched PDB entries containing the compound IDs (_chem_comp.id) of HEM, HEA, HEB, HEC, or HEO with a resolution of 2.0 Å or less using SQL in the PDBj Mine relational database [37] (https://pdbj.org/rdb/search, accessed on 6 October 2022). The PDBx/mmCIF files were downloaded from the Protein Data Bank Japan (PDBj) [38]. Structural information was extracted from the atom_site category of the PDBx/mmCIF file. We collected only one model for each PDB entry. When the occupancy value is <1.0 and pdbx_PDB_model_num is 1, the atom with the largest occupancy was selected from the atoms with the same auth_seq_id and label_asym_id in the atom_site category. When the occupancy was 0.5, we chose the atoms with the label_alt_id of A. This selection was applied even to atoms with different auth_seq_id values in the atom_site category. After collecting the atomic coordinates, we excluded heme molecules missing one or more of the 25 heavy atoms forming the Fe–porphyrin skeleton (Figure 1). Consequently, 6866 heme molecules from 3206 unique PDB entries were obtained. The Bio.PDB package [39] for BioPython version 1.78 [40] was used to parse the mmCIF files.

As a first step in elucidating the correlation between the tertiary structure of an active site and protein function, we used only the structures in which amino acids or water molecules were axially coordinated to heme. Here, 5185 samples were obtained. Axial ligands were defined as amino acid residues or other molecules, including one or more atoms within 3.1 Å of the heme iron atom. MDTraj library version 1.9.5 [41] was used to analyze the structural data. To reduce the redundancy of amino acid sequences, we excluded protein chains with sequence similarity higher than 99.99% using the PISCES server [42]. Finally, the samples in which the coverage of heme was less than 0.6 were excluded because the biological and asymmetric units were likely to differ. This nonredundant dataset was composed of 1234 samples and is referred as dataset_99. Although the oxidation state of Fe is closely related to the protein function, we did not consider it in this study because the aim of this study was an elucidation of the structure–function relationship in hemeproteins and a construction of functional predictor from the pocket structure for this purpose.

### 2.2. Assignment of Protein Function to Each Heme Sample

Information about protein function was assigned by the enzyme commission (EC) number and gene ontology (GO) associated with each entity in each PDB entry, as well as keywords and descriptions stored in each PDB entry. The EC number, GO, keywords, and description were collected by a SQL search from EC_number of sifts.pdb_chain_enzyme table, GOID of gene_ontology_pdbmlplus table, keywords of brief_summary table, and pdbx_description of entity table in PDBj Mine relational database (accessed on 6 October 2022), respectively. First, we assigned function(s) to each sample of the non-redundant dataset as follows:

(1) If the protein chain(s), including the axial ligand(s), had an EC number(s), the first digit of the EC number(s) was assigned.
(2) If case 1 did not apply and the protein chain(s) had GO associated with “oxygen-binding”, “oxidoreductase activity”, “electron transfer activity”, “transcription”, or “heme transport” as the molecular function or biological process, one function was assigned in order from these functions.
(3) If cases 1 and 2 did not apply and the PDB entry had keywords associated with “hemophore”, “electron transfer activity”, “oxygen-binding”, “oxidoreductase activity”, “heme extraction”, “signaling protein”, “nitrophorin (NO transport)”, or “heme transport”, one function was assigned in order from these functions.
(4) If cases 1–3 did not apply and the PDB entry had a description of cytochrome p460, “oxidoreductase” was assigned.
(5) If cases 1–4 did not apply or there was no axial ligand, “unclassified” was assigned.

At this stage, 16 types of function labels, including multi-function combinations, were assigned. Next, we manually modified the function of dehaloperoxidase and myoglobin with oxidoreductase activity to “oxygen-binding and oxidoreductase” (dual-function). In this study, we only used the samples assigned “oxygen-binding”, “oxidoreductase”, “electron transfer”, or “oxygen-binding and oxidoreductase” as protein functions. These protein functions are listed in SI (pdbid_function_list.csv).

### 2.3. CNN Model

**Figure 2:**
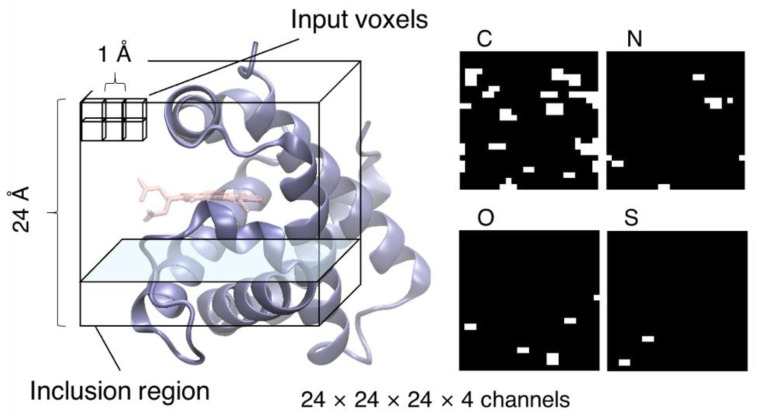
Schematic of the input of our CNN model. The protein backbone and heme molecule are represented as a blue cartoon and a licorice model colored in salmon, respectively. The input voxels were prepared for each atom of C, N, O, and S, as illustrated in the right panel. The coordinates of heme and molecules other than protein were not used in the voxel calculation.

Here, we constructed a CNN model whose input and output were the tertiary structure of the heme-binding pocket and the protein function, respectively. To use the non-uniform structural data of heme-binding site as input for the CNN model, we converted the data into uniform dimensional data. Then, we used voxel sets included in a cube-shaped inclusion region on the heme-binding site as an input (Figure 2). This inclusion region was defined as described below. First, we calculated a least-squares plane for CHA, CHB, CHC, and CHD atoms in the porphyrin ring of heme and defined it as the xy-plane. Then, we rotated the xy-plane such that the x-axis was parallel to the vector connecting CHA and CHC projected onto the least-squares plane and determined the z-axis to be perpendicular to the xy-plane and right-handed. Finally, the origin was translated to the barycenter of CHA, CHB, CHC, and CHD. The edge length of the inclusion region was set to 24 Å, which is identical to the value determined in our previous study [28]. For voxelization, we divided the space included in the inclusion region into the small cubic region (voxel) with an edge length of 1 Å. Using atomic coordinates of protein without heme and molecules other than proteins, we assigned 1 (occupied) or 0 (unoccupied) to each voxel depending on whether it was occupied by any atom or not, respectively. The input voxels were prepared for each atom of C, N, O, and S, and used as an input with four channels. For the detailed procedures for determining the inclusion region and voxelization, please refer to our previous study [28].

The output of the CNN model is a class label of the protein function. Class labels are two- or three-dimensional, allowing multiple functions to be assigned to a single sample. The loss was calculated as binary cross-entropy between the observed (assigned function) and predicted class labels.

We constructed and trained all CNN models using PyTorch version 1.11.0 [43]. The parameters of our CNN model are shown in Table 1. These parameters are identical to those determined in our previous study [28] except the last layer. A brief demonstration regarding the method used in CNN is also described there. The network model was constructed so that the number of layers would not be too large, and the other hyperparameters were roughly tuned. The output dimension of each layer was determined by the number of output channels specified in Convolution layer, and the parameters such as the kernel size and/or stride of the Convolution and Pooling layers. These hyperparameters were set to those commonly used. We tried a couple of models with different hyperparameters for this study, which resulted in almost no effect on accuracy. For training, the stochastic gradient descent optimizer with a learning rate of 0.01 was used, and the batch size was set to 32. To verify the generalization performance of the model, five-fold cross-validation was performed. We did not separate the test and cross-validation datasets because of limited data. The detailed procedure of the cross-validation has been described in our previous study [28].

### 2.4. Analyses of Cavity of Heme-Binding Site

We computed the cavity shapes of heme-binding sites using POVME 3.0 [44]. With POVME, the cavity shape of a ligand-binding pocket can be represented as a bit vector, each element of which represents whether or not the respective grid is located in a ligand-binding cavity, 1 for a cavity and 0 for protein atoms. We refer to this bit vector as a “cavity vector” in the following. To compare the cavity shapes of various proteins, the region to be analyzed was limited to the vicinity of the heme molecule: the center and radius of the inclusion sphere (parameters for POVME) were set to the coordinates of the heme iron atom and 8.5 Å, respectively. We set the grid size to 1 Å and did not use the option for removing isolated points that were not contiguous with the specified region. The detailed procedure for preparing the input protein coordinates has been described in our previous work [28].

## 3. Results and Discussion

### 3.1. Prediction of Protein Function from the Tertiary Structure of the Heme-Binding Pocket Using a CNN Model: Two-Label Classification

We constructed a CNN model to predict the function of proteins classified into the following three classes, namely, oxygen-binding protein (OB), oxidoreductase (OR), and proteins with both functions (OB–OR), from the tertiary structures of heme-binding pockets by using the dataset_99. The output of the CNN model is two-dimensional, with each label indicating whether each function (oxygen-binding or oxidoreductase) is retained, namely, (0, 1), (1, 0), and (1, 1) represent the OB, OR, and OB–OR classes, respectively. Only when the values of the two labels matched between the observed and predicted ones were the results considered true positives (TP). The obtained models were evaluated in terms of the score, $S_{\mathrm{acc}}$, calculated as follows:

(1) $$S_{\mathrm{acc}}=\frac{\sum_{c\in L}N_{c}^{\mathrm{TP}}}{\sum_{c\in L}N_{c}}$$

where *L*, $N_{c}$, and $N_{c}^{\mathrm{TP}}$ represent the labels of function, the number of samples belonging to class *c*, and the number of samples in class *c* that are TP as a result of prediction, respectively. In this analysis, *L* = {OB, OR, OB–OR}. $N_{\mathrm{OB}}$, $N_{\mathrm{OR}}$, and $N_{\mathrm{OB}-\mathrm{OR}}$ for the test sets of five-fold cross-validation runs were 190, 312, and 35, respectively. The mean and standard deviation of the $S_{\mathrm{acc}}$ scores obtained from five-fold cross-validation was 0.959 ± 0.021, indicating high prediction accuracy.

We also calculated the confusion matrix **M** using the scikit-learn Python library [45] version 0.24.2 (Table 2). The non-diagonal element of a confusion matrix, **M***_ij_*, represents the actual number of observations in class *i* but are predicted to be in class *j*. The confusion matrix of Table 2 was normalized, and each element has a mean value over five cross-validation runs. Although in two-label classification, the predicted value can also be (0, 0), which means that the sample is neither OB nor OR, there was no sample with a predicted value of (0, 0) in this analysis. Therefore, such a sample is omitted in Table 2. The protein function could be predicted with very high accuracy for the single-function proteins (OB and OR). However, protein function prediction was difficult for the dual-function proteins (OB–OR). We also calculated the mean values of accuracy, recall, precision, and specificity over the five-fold cross-validation runs for each class (Table S1). For the calculation of these indicators, we defined **M***_ii_* as TP, M*_ji_* (j ≠ i) as false positive, **M***_ij_* (j ≠ i) as false negative, and **M***_jk_* (*j* ≠ *i*, *k* ≠ *i*) as true negative for class *i*. Whereas all indicators were high in the single-function proteins, only precision was high in the OB–OR. The latter means that samples that were predicted to be OB–OR were correct, but there were many samples belonging OB–OR that could not be correctly predicted. The dual-function proteins contain two types of proteins: dehaloperoxidase and myoglobin mutants. The ratios of TP in the samples included in the test sets of five cross-validation runs were 1.0 (9/9) for dehaloperoxidase and 0.423 (11/26) for myoglobin mutants with a dual-function (DF-Myoglobin). The low TP rate in the OB–OR class was due to the inaccuracy of the prediction of the function of DF-Myoglobins. Considering that the dataset_99 includes 116 samples with the description of “myoglobin” in PDB, 32 of which have dual functions, it is likely that the prediction was influenced by samples with similar pocket structures but a different function.

Next, we examined in detail the samples with inaccurate function prediction. Fifteen of the twenty-one samples with inaccurate predictions were DF-Myoglobins, most of which were predicted to belong to the OB class. The samples other than DF-Myoglobins classified as OB are listed in Table 3. PDB ID of 3QZX [46] is protoglobin, which has highly distorted heme, suggesting that the pocket structure is different from those of other oxygen-binding proteins. For PDB IDs of 3QZX, 4XDI [47], and 6O0A [48], there was no sample with a similar amino acid sequence (similarity ≥ 0.7). The lack of sufficient training data may be the cause of prediction failure. For two cases (PDB ID of 2BK9 [49] and 3MVC [50]), the protein function assignment may be wrong, and the predicted results were correct (misassignment of protein function). Although the former is hexacoordinate hemoglobin, which is expected to function as oxidoreductase, it is unclear whether this protein exhibits enzymatic activity. The latter exhibits oxidoreductase activity and no affinity to the oxygen molecules, but OB was assigned as the protein function. There was a sample with OB as the class label (observed value) and OB–OR as the predicted value (PDB ID: 7CEZ). PDB ID of 7CEZ is myoglobin G5K/Q8K/A19K/V21K mutant. Its functional property is unknown because the paper is unpublished. This mutant may exhibit oxidoreductase activity, as we predicted. Considering these results, protein function assignment is one of the significant challenges in this type of research.

### 3.2. Specification of Regions in Input Data Significant for Prediction

**Figure 3:**
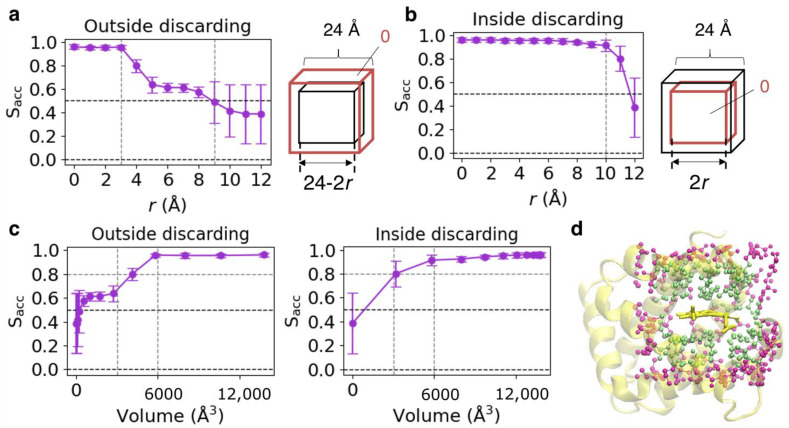
(**a**) Mean $S_{\mathrm{acc}}$ scores plotted against *r*, which is the distance between the faces of the outer (red) and inner (black) cubes presented in the right panel for “outside discarding.” The error bar shows the standard deviation. The centers of the outer and inner cubes are identical, and their edges are parallel. (**b**) Mean $S_{\mathrm{acc}}$ scores plotted versus *r* for ”inside discarding.” (**c**) $S_{\mathrm{acc}}$ scores versus the volume of the region with the original information. (**d**) Atoms included in cube-shaped regions with an edge length of *l* are illustrated using the PDB entry of 1A00 as an example. The lime spheres and the combination of lime and magenta spheres represent *l* = 18 and 24, respectively. The main chain of the host protein is shown as a yellow cartoon, and heme as a yellow stick.

To determine the regions significant for predicting protein function, we examined the change in prediction scores when information about a specific region of input voxels was discarded. The model constructed in Section 3.1 was used for this analysis. Information was discarded in two ways. We refer to them as “outside discarding” and “inside discarding”, which remove information from the outside (Figure 3a) and inside (center) (Figure 3b), respectively. First, two cubes were defined: the “outer cube” and the “inner cube”. The vertex coordinates of the outer cube are (±12, ±12, ±12), being equivalent to the inclusion region of the CNN model. Let the vertex coordinates of the inner cube be (±(12–*r*), ±(12–*r*), ±(12–*r*)) on the “outside discarding” and be (±*r*, ±*r*, ±*r*) on “inside discarding”. Then, the sets of voxels in the outer and inner cubes are denoted as *V*_outer_ and *V*_inner_, respectively. The voxels in *V*_outer_ but not in *V*_inner_ were replaced with 0 for “outside discarding” (0 ≤ *r* < 12, Figure 3a), and those of *V*_inner_ were replaced with 0 for “inside discarding” (0 ≤ *r* < 12, Figure 3b). In both cases, information is intact (not discarded) at *r* = 0.

$S_{\mathrm{acc}}$ for “outside discarding” and “inside discarding” averaged over the test sets in the five-fold cross-validation runs are presented in the left panels of Figure 2a,b, respectively. Because the amount of information loss on the *r* value was different between “outside discarding” and “inside discarding” and nonlinear, $S_{\mathrm{acc}}$ scores were also plotted against the volume of the region with the original information (Figure 3c). Considering that the change in $S_{\mathrm{acc}}$ scores between the volumes of 3000 and 6000 Å^3^ differed for “outside discarding” and “inside discarding,” the score would depend on the region used for prediction. Whereas the scores dropped sharply when the value of *r* exceeded 3 Å, where the edge length of the inner cube was 18 Å and reached almost 0.5 *r* = 9 Å in “outside discarding”, it did not significantly change between the values of *r* from 0 to 10 Å, where the edge length of the inner cube is 0–20 Å in “inside discarding.” These results suggest that the prediction was performed using the information near the surface of the outer cube (input voxels). Examples of *A_l_* (*l* = 18 and 24), which is an atom set included in the cube with edge lengths of *l*, are illustrated in Figure 3d using a PDB entry of 1A00. This may be one of the reasons why it was difficult to distinguish amino acid mutations in the heme-binding pocket of DF-Myoglobin. We also constructed a CNN model using smaller input voxels (edge length = 17 Å) as an input. However, almost the same result was obtained (the mean and standard deviation of $S_{\mathrm{acc}}$ score over five-fold cross-validation was 0.959 ± 0.024). The confusion matrix is shown in Table S2. The modification of inputs may be required to incorporate information about the pocket surface into the prediction.

### 3.3. Prediction of Protein Function from the Tertiary Structure of the Heme-Binding Pocket Using a CNN Model: Three-Label Classification

We constructed a CNN model with three-dimensional output to predict the functions of proteins classified into the following four classes: OB, OR, OB–OR, and electron transport protein (ET) by using the dataset_99. Other classes were not assigned in this study. The output is three-dimensional, with each label indicating whether or not each function (oxygen-binding, oxidoreductase, or electron transfer) is retained, namely, (0, 1, 0), (1, 0, 0), (1, 1, 0), and (0, 0, 1) represent the OB, OR, OB–OR, and ET classes, respectively. Only when the values of the three labels matched between the observed and predicted ones were the results considered TP.

The number of samples belonging to OB, OR, OB–OR, and ET for the test sets of five-fold cross-validation were 193, 297, 36, and 371, respectively. The prediction accuracy was also reasonably high in the three-label classification, and the mean and standard deviation of the $S_{\mathrm{acc}}$ for *L* = {OB, OR, OB–OR, ET} in Equation (1) obtained from the five-fold cross-validation were 0.895 ± 0.031. As shown in the confusion matrix shown in Table 4, while the recall for the OB class was as high as that in the two-label classification, that for OR became lower and was nearly the same as that for ET. This may be because of the functional similarity between OR and ET. We also calculated the mean values of accuracy, recall, precision, and specificity over the five-fold cross-validation runs for each class (Table S3). Some of the samples that were erroneously predicted as ET despite being OR had a keyword associated with “electron transfer” in PDB. Notably, the low false recognition rates between OB and ET and between OB and OR, suggest a clear difference in the tertiary structures of their active sites. This indicates the structure–function relationships in the active sites of heme proteins. We expect the application of this method to the classification of a wider variety of protein functions in the future.

### 3.4. Validation of Datasets Used for CNN Model Construction

To validate the dataset used for the CNN model construction in this study, we constructed CNN models using the additional datasets with different thresholds of the sequence similarity. Although a previous study, in which the heme-binding site was detected from the property of pocket cavity, adopted a threshold of 80% [36], a sufficient value of the threshold of sequence similarity is generally debatable [51]. Here, we used 25.00, 60.00, 80.00, and 99.99% as the threshold of sequence identity for nonredundant datasets. This is because thresholds of 25% were adopted for the prediction of secondary structure [52] and disorder region [53], and a threshold of 60% of the motif length was proposed for the prediction of post-translational modifications [54]. Since these datasets included few samples of OB-OR, we removed the OB-OR samples from each dataset and carried out the classification of OB and OR (two-label and two-class classification). We referred to these datasets as dataset_25, dataset_60, dataset_80, and dataset_99_without_OB-OR, respectively, in the following. The mean $S_{\mathrm{acc}}$ scores over five-fold cross-validation runs were 0.923 ± 0.069, 0.934 ± 0.089, 0.974 ± 0.022 and 0.990 ± 0.011 for the dataset_25, dataset_60, dataset_80, and dataset_99_without_OB-OR, respectively. The mean values of accuracy, recall, precision, and specificity over five-fold cross-validation runs are listed in Table 5. Despite the bias in the sample numbers of each class, most indicators showed high values in both classes even in the dataset of dataset_25.

In addition, we performed the same analysis of Section 3.2 with the CNN model constructed by the dataset_25. As shown in Figure S1, the behaviors of both “outside discarding” and “inside discarding” are similar to those of the dataset_99, suggesting that both networks by the dataset_25 and dataset_99 may use similar features.

We also constructed a CNN model by using the dataset_25 for three-label classification, the same analysis as Section 3.3, and obtained the mean $S_{\mathrm{acc}}$ score of 0.767 ± 0.083. The number of samples belonging to OB, OR, and ET for the test sets of five-fold cross-validation were 15, 54, and 31, respectively. There was a sample that was erroneously classified as Others. The confusion matrix and values of accuracy, recall, precision, and specificity were listed in Tables S4 and S5. The slight decrease in the mean $S_{\mathrm{acc}}$ score compared with that of the dataset_99 would be mainly due to misclassification of OR. There was an increase in the number of cases where the OB was classified as OR and the OR was classified as ET. The small sample number may lead to a decrease in accuracy with an increase in class labels.

These results indicate that the presence of similar data does not unfairly increase accuracy, namely, the effect of a large value of the sequence identity is small. A similar kind of robustness to the sequence identity cutoff has been demonstrated for the performance of a structure-based graph convolution network model over the function prediction [55]. Therefore, we conclude that the sequence homology would have little impact on our problem.

### 3.5. Similarity of the Structures of Heme-Binding Pockets between Proteins with the Same Function

To estimate the similarity of cavity shapes of the heme-binding sites in proteins with the same function, we analyzed the variability of cavity shapes for each protein group using cavity vectors computed by POVME software. Let *I* be a set of samples of cavity shapes in a protein group. The mean distance from the barycenter for cavity vector ***v****_i_* was calculated for each protein group as an indicator of dispersion of a set of cavity vectors following the same procedure as our previous work [28], as follows:

(2) $$N_{I}=\left|I\right|(the number of samples ofI),$$

(3) $$\mu_{I}=\frac{1}{N_{I}}\sum_{i\in I}v_{i}$$

(4) $${\bar{d}}_{I}=\frac{1}{N_{I}}\sum_{i\in I}||v_{i}-\mu_{I}||$$

where || || represents the *L*^2^ norm.

The protein group identifier, number of samples, and ${\bar{d}}_{I}$ for each protein group calculated for the dataset_99 and dataset_25 are shown in Table 6. Results for the combined group of OB, OR, and OB–OR (referred to as “Combined” in the following), dehaloperoxidase, DF-Myoglobin, and myoglobin (OB) are also listed for comparison. For smaller ${\bar{d}}_{I}$ values, higher cavity shape similarity was expected in a protein group.

As shown in Table 6, similar results were obtained for the dataset_99 and dataset_25. While ${\bar{d}}_{I}$ was slightly small in the OB and OB–OR classes for the result of the dataset_99, it was as high as that in the “Combined” group, including four protein groups for the OR and ET classes. For homologous protein groups, dehaloperoxidase and myoglobin, ${\bar{d}}_{I}$ was significantly smaller than that of the “Combined” group. The ${\bar{d}}_{I}$ of DF-Myoglobin was slightly larger than that of myoglobin, suggesting that the mutations in the active site change the cavity structures. This implies that the structure of an active site is not similar among proteins with the same function but varies significantly among protein groups. Considering the results of Section 3.1, the proteins with the same function have a common structural feature in spite of the difference in the overall cavity shapes.

## 4. Conclusions

In this study, we constructed a CNN model to predict protein functions from the tertiary structures of the active sites of heme proteins to examine the structure–function relationship. High $S_{\mathrm{acc}}$ scores (>0.95) were obtained by the CNN model for two-label classification for classifying OB, OR, and OB–OR. There were a few cases of false positives due to the misassignment of protein function, i.e., the predicted results were correct, resulting in the issue of improving the method of function assignment. In addition, the prediction of the function of engineered myoglobin (functionally modified mutants) remained a challenge. Because myoglobin is mostly an oxygen carrier, the difficulty in predicting the function of functionally modified mutants may be due to the lack of sufficient data. The analysis results of the similarity of cavity shape among proteins with the same function indicate that there is no one-to-one correspondence between the protein function and pocket structure, suggesting that the proteins with the same function have a common structural feature in spite of the difference in the overall cavity shapes. Predicting the modified function of proteins with a single amino acid mutation may require some ingenuity.

We also constructed a CNN model for three-label classification to classify OB, OR, OB–OR, and ET. Although the overall accuracy was slightly lower than that of the two-label classification, the recall for OB was maintained at the same level as that for the two-label classification. The misclassification between OB and ET and between OB and OR is almost zero, indicating that the prediction works well between the groups with different functions. The application of this study to classification tasks with more labels is expected.

Overall, this study demonstrated the structure–function correlation in the active sites of heme proteins. In the future, we will attempt to construct a model to predict more detailed protein functions, such as catalytic reactions or function of proteins binding heme as a non-active center, such as hemophores. To improve the accuracy and robustness of the CNN model, we will attempt to increase the amount of structural data, improve the function assignment method, modify the input information, and so on. Since the protein dynamics are also important for protein function, we will also attempt to include them into the input to improve our CNN model in the future. Our previous study showed that AlphaFold2 [56], which is a deep learning algorithm for predicting the tertiary structure of proteins from the amino acid sequence, can accurately predict the structure of the heme-binding site in heme proteins [57]. If the challenge of predicting heme-binding sites from their amino acid sequences could be overcome, protein functions would be predicted using their amino acid sequences for heme proteins. We would like to attempt this challenge in the future.

## Figures and Tables

**Table 1:** Layers and parameters of our CNN model.

Layer	Function	Filter (Kernel)	Output Dimension (Channel × Depth × Width × Height)
1	Conv3d	2 × 2 × 2 with 0-padding	64 × 21 × 21 × 21
2	Conv3d	2 × 2 × 2 with 0-padding	128 × 22 × 22 × 22
3	BatchNorm3d	-	128 × 22 × 22 × 22
4	Conv3d	2 × 2 × 2 without padding	128 × 21 × 21 × 21
5	ReLU	-	128 × 21 × 21 × 21
6	BatchNorm3d	-	128 × 21 × 21 × 21
7	MaxPool3d	2 × 2 × 2 stride: 2 × 2 × 2	128 × 10 × 10 × 10
8	Full connection	-	128,000
9	Linear	-	128
10	ReLU	-	128
11	Dropout	0.4	128
12	Linear	-	64
13	BatchNorm1d	-	64
14	ReLU	-	64
15	Linear	-	2 or 3
16	Sigmoid	-	2 or 3

**Table 2:** Mean values and standard deviations of the normalized confusion matrices over five-fold cross-validation runs. Values in the parentheses represent the confusion matrix calculated with the combined data of the test sets of five-fold cross validation runs for two-label classification.

		Predicted Value		
		OB	OR	OB–OR
**Observed Value**	**OB [190] ^†^**	0.985 ± 0.012 (187)	0.010 ± 0.012 (2)	0.005 ± 0.010 (1)
	**OR [312] ^†^**	0.010 ± 0.008 (3)	0.990 ± 0.008 (309)	0.000 ± 0.000 (0)
	**OB–OR [35] ^†^**	0.436 ± 0.248 (15)	0.000 ± 0.000 (0)	0.564 ± 0.248 (20)

**^†^** Values in the square brackets represent the sample numbers of each class.

**Table 3:** List of the samples that failed to predict other than those classified as OB and DF-Myoglobins.

PDB ID	Protein Name	Observed Value	Predicted Value	Remark
2BK9	hemoglobin	OR	OB	misassignment
3MVC	GLB-6	OB	OR	misassignment
3QZX	protoglobin	OB	OR	-
4XDI	THB1 (truncated hemoglobin)	OR	OB	-
6O0A	flavohemoglobin	OR	OB	-
7CEZ	myoglobin (G5K/Q8K/A19K/V21K)	OB	OB–OR	detailed function unknown

**Table 4:** Mean values and standard deviations of the normalized confusion matrices over five cross-validation runs. Values in the parentheses represent the confusion matrix calculated with the combined data of the test sets of five-fold cross validation runs for three-label classification.

		Predicted Value				
		OB	OR	OB–OR	ET	Others ^†^
**Observed Value**	**OB [193] ^‡^**	0.973 ± 0.016 (188)	0.016 ± 0.013 (3)	0.006 ± 0.012 (1)	0.000 ± 0.000 (0)	0.005 ± 0.010 (1)
	**OR [297] ^‡^**	0.006 ± 0.007 (2)	0.907 ± 0.054 (268)	0.000 ± 0.000 (0)	0.084 ± 0.049 (26)	0.004 ± 0.007 (1)
	**OB–OR [36] ^‡^**	0.570 ± 0.296 (20)	0.000 ± 0.000 (0)	0.430 ± 0.296 (16)	0.000 ± 0.000 (0)	0.000 ± 0.000 (0)
	**ET [371] ^‡^**	0.000 ± 0.000 (0)	0.110 ± 0.019 (40)	0.000 ± 0.000 (0)	0.890 ± 0.019 (331)	0.000 ± 0.000 (0)

**^†^** ”Others” represents the predicted value of (0, 0, 0). **^‡^** Values in the square brackets represent the sample numbers of each class.

**Table 5:** Mean values and standard deviations of accuracy, precision, recall, and specificity obtained from two-label classification over the five-fold cross-validation runs for each class.

Dataset	Class Label	Accuracy	Recall	Precision	Specificity
Dataset_25	OB [9] ^†^	0.923 ± 0.069	0.800 ± 0.400	0.653 ± 0.366	0.925 ± 0.074
	OR [55] ^†^	0.923 ± 0.069	0.925 ± 0.074	0.983 ± 0.033	0.800 ± 0.400
Dataset_60	OB [36] ^†^	0.934 ± 0.089	0.703 ± 0.381	0.979 ± 0.036 ^‡^	0.994 ± 0.013
	OR [192] ^†^	0.934 ± 0.089	0.994 ± 0.013	0.937 ± 0.090	0.703 ± 0.381
Dataset_80	OB [59] ^†^	0.974 ± 0.022	0.932 ± 0.097	0.896 ± 0.106	0.983 ± 0.015
	OR [239] ^†^	0.974 ± 0.022	0.983 ± 0.015	0.987 ± 0.017	0.932 ± 0.097
Dataset_99_ without_OB-OR	OB [196] ^†^	0.990 ± 0.011	0.995 ± 0.010	0.981 ± 0.026	0.987 ± 0.020
	OR [308] ^†^	0.990 ± 0.011	0.987 ± 0.020	0.997 ± 0.007	0.995 ± 0.010

^†^ Values in the square brackets represent the sample numbers of the test sets of each class. ^‡^ The results averaged over four runs of the five-fold cross-validation runs because both TP and FP were 0 in a run.

**Table 6:** Protein groups, sample numbers, and ${\bar{d}}_{I}$. The shaded row represents the protein group combined OB, OR, OB–OR, and ET.

Protein Group	Sample Number		${\bar{d}}_{I}$	
	Dataset_99	Dataset_25	Dataset_99	Dataset_25
OB	241	16	12.70 (1.78) ^†^	15.45 (2.23) ^†^
OR	388	63	16.88 (2.45) ^†^	15.86 (2.61) ^†^
OB–OR	42	0	12.44 (2.41) ^†^	-
ET	450	47	16.52 (2.88) ^†^	16.07 (2.05) ^†^
Combined	1121	126	16.80 (2.51) ^†^	15.99 (2.34) ^†^
Dehaloperoxidase	10	0	9.95 (1.21) ^†^	-
DF-Myoglobin	32	0	11.20 (2.60) ^†^	-
Myoglobin (OB)	82	1	9.99 (2.33) ^†^	0.00 (0.00) ^†^

^†^ Values in parentheses represent the standard deviation of $||v_{i}-\mu_{I}||.$

## Data Availability

The atomic coordinates of heme proteins were downloaded from PDBj (https://pdbj.org/, accessed on 6 October 2022). For convenience, the list of PDB IDs and assigned protein functions is provided in Supplementary Materials.

## Supplementary Materials

The following supporting information can be downloaded at: https://www.mdpi.com/article/10.3390/biom13010137/s1, Table S1: Confusion matrix resulted from the two-label classification with the edge length of inclusion region of 12.0 Å; Table S2: Confusion matrix resulted from the two-label classification with the edge length of inclusion region of 8.5 Å; Table S3: Mean values and standard deviations of accuracy, precision, recall, and specificity obtained from three-label classification; Figure S1: Plots of mean $S_{\mathrm{acc}}$ scores of the outside discarding and inside discarding for the CNN model by using the dataset_25; Table S4: Mean values and standard deviations of the normalized confusion matrices for three-label classification with the dataset_25; Table S5: Mean values and standard deviations of precision, recall, and specificity obtained from three-label classification by using the dataset_25.
