# Supplementary material for: Prediction of Protein Function from Tertiary Structure of the Active Site in Heme Proteins by Convolutional Neural Network
Source: Biomolecules. 2023 Jan 9;13(1):137. doi: 10.3390/biom13010137 (PMC9855806; doi:10.3390/biom13010137)
Supplement: Supplementary file 1 [file biomolecules-13-00137-s001.zip › SI.pdf]

Prediction of Protein Function from Tertiary Structure of the Active Site in Heme Proteins by Convolutional Neural Network

Hiroko X. Kondo, Hiroyuki Iizuka, Gen Masumoto, Yuichi Kabaya, Yusuke Kanematsu, and Yu Takano

Table S1. Mean values and standard deviations of accuracy, precision, recall, and specificity obtained from two-label classification over the five-fold cross validation runs for each class with the edge length of inclusion region of 12.0 Å.

	Accuracy	Recall	Precision	Specificity
**OB [190]** †	0.961 ± 0.019	0.985 ± 0.012	0.914 + 0.043	0.948 ± 0.030
**OR [312]**	0.991 ± 0.006	0.990 ± 0.008	0.993 ± 0.008	0.992 ± 0.010
**OB-OR [35]**	0.970 ± 0.015	0.564 ± 0.248	0.975 ± 0.005	0.998 ± 0.004

† Values in the square brackets represent the sample numbers of the test sets of each class.

Table S2. Mean values and standard deviations of the normalized confusion matrices over five-fold cross-validation runs with the edge length of inclusion region of 8.5 Å. Values in the parentheses represent the confusion matrix calculated with the combined data of the test sets of five-fold cross-validation runs of two-label classification.

		**Predicted Value**			
		**OB**	**OR**	**OB-OR**	**Others** †
**Observed Value**	**OB [190]** ‡	0.990 ± 0.012	0.010 ± 0.012	0.000 ± 0.000	0.000 ± 0.000
		(188)	(2)	(0)	(0)
	**OR [312]**	0.010 ± 0.008	0.984 ± 0.014	0.003 ± 0.006	0.003 ± 0.006
		(3)	(307)	(1)	(1)
	**OB-OR [35]**	0.436 ± 0.248	0.000 ± 0.000	0.564 ± 0.248	0.000 ± 0.000
		(15)	(0)	(20)	(0)

† "Others" represents the predicted value of (0, 0). ‡ Values in the square brackets represent the sample numbers of the test sets of each class.

Table S3. Mean values and standard deviations of accuracy, precision, recall, and specificity obtained from three-label classification over the five-fold cross-validation runs for each class by using the dataset_99. Values in the square brackets represent the sample numbers of the test sets of each class.

	Accuracy	Recall	Precision	Specificity
**OB [193]**	0.970 ± 0.013	0.973 ± 0.016	0.896 + 0.054	0.969 ± 0.017
**OR [297]**	0.920 ± 0.020	0.907 ± 0.054	0.860 ± 0.012	0.928 ± 0.009
**OB-OR [36]**	0.977 ± 0.012	0.430 ± 0.296	0.938 ± 0.108 †	0.999 ± 0.002
**ET [371]**	0.926 ± 0.019	0.890 ± 0.019	0.924 ± 0.041	0.952 ± 0.028

† The results averaged over four runs of the five-fold cross-validation runs because both TP and FP were 0 in a run.

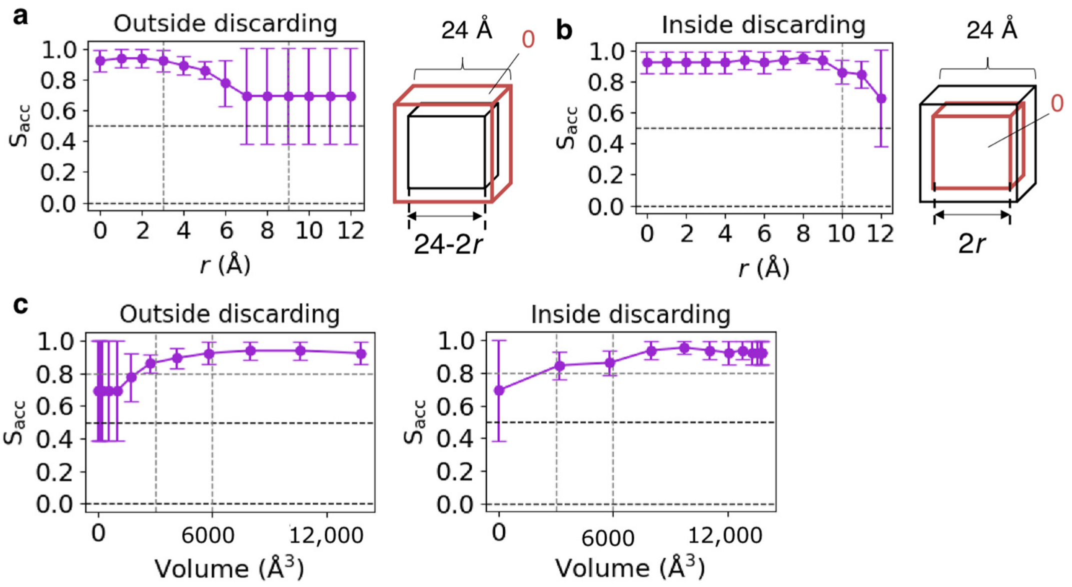

Figure S1. (a) Mean S_{acc} scores plotted against r, which is the distance between the faces of the outer (red) and inner (black) cubes presented in the right panel for "outside discarding." The error bar shows the standard deviation. The centers of the outer and inner cubes are identical, and their edges are parallel. (b) Mean S_{acc} scores plotted versus r for "inside discarding." (c) S_{acc} scores versus the volume of the region with the original information.

Table S4. Mean values and standard deviations of the normalized confusion matrices over five cross-validation runs. Values in the parentheses represent the confusion matrix calculated with the combined data of the test sets of five-fold cross validation runs for three-label classification.

		**Predicted Value**			
		**OB**	**OR**	**ET**	**Others** †
**Observed Value**	**OB [15]** ‡	0.893 ± 0.137 (12)	0.107 ± 0.137 (3)	0.000 ± 0.000 (0)	0.000 ± 0.000 (0)
	**OR [54]**	0.060 ± 0.054 (4)	0.743 ± 0.155 (38)	0.183 ± 0.100 (11)	0.014 ± 0.029 (1)
	**ET [31]**	0.000 ± 0.000 (0)	0.092 ± 0.130 (3)	0.908 ± 0.130 (28)	0.000 ± 0.000 (0)
	**Others [0]**	0.000 ± 0.000 (0)	0.000 ± 0.000 (0)	0.000 ± 0.000 (0)	0.000 ± 0.000 (0)

† "Others" represents the predicted value of (0, 0, 0). ‡ Values in the square brackets represent the sample numbers of the test sets of each class.

Table S5. Mean values and standard deviations of precision, recall, and specificity obtained from three-label classification over the five-fold cross-validation runs for each class by using the dataset_25.

	**Accuracy**	**Recall**	**Precision**	**Specificity**
**OB [15]**	0.930 ± 0.023	0.893 ± 0.137	0.700 + 0.267	0.959 ± 0.038
**OR [54]**	0.785 ± 0.101	0.743 ± 0.155	0.864 ± 0.075	0.869 ± 0.094
**ET [31]**	0.864 ± 0.100	0.908 ± 0.130	0.736 ± 0.175	0.845 ± 0.097
